# Alterations in the Urinary Microbiota Are Associated With Cesarean Delivery

**DOI:** 10.3389/fmicb.2018.02193

**Published:** 2018-09-12

**Authors:** Fengping Liu, Longxian Lv, Huiyong Jiang, Ren Yan, Shurong Dong, Liping Chen, Wei Wang, Yong Q. Chen

**Affiliations:** ^1^Wuxi School of Medicine, Jiangnan University, Wuxi, China; ^2^Collaborative Innovation Center for Diagnosis and Treatment of Infectious Diseases, State Key Laboratory for Diagnosis and Treatment of Infectious Diseases, The First Affiliated Hospital, School of Medicine, Zhejiang University, Hangzhou, China; ^3^Key Laboratory of Advanced Micro/Nano Electronic Devices and Smart Systems of Zhejiang, College of Information Science and Electronic Engineering, Zhejiang University, Hangzhou, China; ^4^Intensive Unit, Affiliated Yancheng Hospital, School of Medicine, Southeast University, Nanjing, China; ^5^Department of Urology, The First Affiliated Hospital, College of Medicine, Zhejiang University, Hangzhou, China

**Keywords:** cesarean section, delivery, *Lactobacillus*, Proteobacteria, urinary microbiota

## Abstract

Similar to the gut, the bladder contains urinary microbiota, and its bacterial composition and structure are determined by the individual’s health status. Cesarean section is a traumatic event for women and it is correlated with postpartum complications. To better understand the urinary microbiota alterations caused by cesarean section, 16S rDNA sequencing was used to assess urine specimens collected by transurethral catheterization from 30 healthy women undergoing cesarean section pre-delivery (PreD) and post-delivery (PostD). A significant increase in bacterial diversity and more detectable bacteria at the phylum, family, and genus levels was observed in the PostD group compared to the PreD group, indicating that cesarean delivery (a process that includes surgery and delivery) altered the bacterial community. Specifically, the phylum Firmicutes and its affiliated family Lactobacillaceae and genus *Lactobacillus* dramatically decreased in the PostD group, suggesting that beneficial bacteria decreased after cesarean section, and clinicians should be aware that this might increase the risk of complications. Concurrently, the phylum Proteobacteria and its affiliated bacteria Pseudomonadaceae and *Pseudomonas* increased in the PostD group compared to the PreD group. This indicates that pathogen growth increases after cesarean section, making it important for clinicians to combat these changes to protect women from infectious diseases. Interestingly, several metabolic pathways, such as metabolism of energy, cofactors and vitamins were strengthened in the PostD group, whereas membrane transport was lessened in this group. This suggests that women’s metabolic disorders might be cured by balancing urinary microbiota. In conclusion, the altered urinary microbiota between the PreD and PostD periods appears to provide insight into how to prevent postpartum metabolic disorders.

## Introduction

Cesarean sections have risen in frequency in many countries around the world. Globally, the cesarean rate was 12.0% in 2000 and 15.5% in 2012 (Ye et al., 2016), while in developing countries, including China, it was 13.1% in 2000 and 16.9% in 2012 (Ye et al., 2016). Cesarean section is associated with future complications (Zakerihamidi et al., 2015), including hemorrhage, surgical site infections, chest infections, and urinary tract infection (UTI), etc. (Yang and Sun, 2017). UTI and surgical site infections are the two infections that most commonly occur after cesarean delivery (Vincent et al., 2008). The prevalence of postpartum UTI in women who underwent cesarean section was 2.8%, and it was 1.50% in women who underwent vaginal delivery (Leth et al., 2009).

Traditionally, clinicians have always associated microorganisms in an individual’s bladder with UTI. An individual is diagnosed with a UTI or asymptomatic bacteriuria if urine culture shows that an individual bacterial count is >10^5^ CFU/mL (Vasudevan, 2014). However, metagenomic analyses have demonstrated that the female bladder harbors an indigenous microbiota (Thomas-White et al., 2018). Therefore, the presence of bacteria in the bladder does not necessarily indicate UTI. Moreover, the composition of the urinary microbiota is affected by the individual’s health status. For example, Pearce et al. (2014) reported that patients with urgent urinary incontinence had higher abundance of *Gardnerella* and lower abundance of *Lactobacillus*. Thomas-White et al. (2018) found that urgent urinary incontinence patients had increased bacterial diversity compared to healthy controls. Furthermore, bacterial diversity affects patients’ treatment response (Karstens et al., 2016). Siddiqui et al. (2012) noted that patients with interstitial cystitis had lower bacterial diversity, with increased *Lactobacillus*. In a study by Abernethy et al. (2017) interstitial cystitis patients had fewer *Lactobacillus* than healthy females. In addition, they found that the urinary microbiota was affected by the patients’ characteristics. For instance, patients with *Lactobacillus* species had lower Female Genitourinary Pain Index, Interstitial Cystitis Symptom Index, and Interstitial Cystitis Problem Index scores than patients without isolated *Lactobacillus* species (Abernethy et al., 2017).

Recently, researchers have begun to explore the correlations between the urinary microbiota and pregnancy and preterm delivery. Yoo et al. (2016) found that bacteria-derived extracellular vesicles (EVs) excreted via the urinary tract were significantly different between pregnant and non-pregnant women. For example, *Bacillus* spp. EVs predominated in the urine of pregnant women, while *Pseudomonas* spp. EVs predominated in the urine of non-pregnant women (Yoo et al., 2016). Furthermore, pregnant women who underwent normal delivery had different EVs profiles from preterm delivery women, such as *Ureaplasma* spp. and Veillonellaceae EVs being more frequently detected in the urine of preterm delivery women (Yoo et al., 2016). However, in another study conducted by Ollberding et al. (2016) no differences in taxa richness, evenness, and bacterial communities were found in the urine from women with preterm or normal deliveries.

Although alteration in the urinary microbiota is associated with pregnancy and preterm delivery, no study has explored whether cesarean section can lead to dysbiosis of the urinary microbiota or whether the composition of the urinary microbiota affects puerperium complications. Our primary aim was to investigate differences in the urinary microbiota between the pre-delivery (PreD) and post-delivery (PostD) periods.

## Materials and Methods

### Recruitment of Subjects

A self-controlled case series approach was used in the design of the present study. The participants were pregnant women who were going to undergo cesarean section. The present study is the first self-controlled case series on the urinary bacterial community in women undergoing cesarean section. It was impossible to obtain the values for the mean and standard deviation from previous studies, so the appropriate sample size was determined according to a recommendation from Benner, who suggested that the sample size should be > 25 cases (Benner, 1999). At least 28 participants were needed in the present study as approximately 10% of patients have negative sequencing results in urinary microbiota studies (Karstens et al., 2016).

Thirty participants were recruited from the Obstetrics Department of the Sixth People’s Hospital of Hangzhou from April to June 2017. The inclusion criteria were as follows: willingness to participate the study; aged ≥ 18 years; pregnant women who were going to undergo an elective cesarean section; consciousness; no history of iodine allergy; and no fever. The exclusion criteria were as follows: UTI in the past month; use of antibiotics, probiotics, or synbiotics in the past month; current abnormal vaginal discharge or itching; or intestinal cystitis, urinary incontinence, urinary tract deformity, bladder protrusion, hydronephrosis, renal atrophy, neurogenic cystitis, or kidney transplantation.

Before collecting the first urine specimen, data on each participant’s characteristics, such as age, parity, gravidity, vital signs, and medication history, were collected. The Ethics Committee of the First Affiliated Hospital of the School of Medicine at Zhejiang University approved the study (reference number: 413). Written informed consent was obtained from each patient before enrollment.

### Urine Specimen Collection

On the day of the cesarean section, a 50 mL urine specimen was collected in two sterile centrifuge tubes using a 50 mL sterile syringe via an indwelling urinary transurethral catheter while conducting transurethral catheterization before surgery. 40 mL urine was collected in a tube for urinary microbiota sequencing and the remaining urine was collected in another tube for standard urine culture. After 24 h, another 50 mL urine specimen was collected while withdrawing the catheter. Prior to collecting the urine using a sterile syringe, the junction between the catheter and the urine collection bag was disinfected with a poly (vinylpyrrolidone) –iodine antiseptic solution (Dian’erkang, Shanghai, China) twice.

### Urine Specimen Processing

The procedure for urine specimen processing was described in our previous study (Ling et al., 2017). Briefly, the urine specimen was discarded if it was confirmed to be contaminated (based on standard urine culture). Non-contaminated specimens were given an anonymous identification code, and they were then immediately transferred to the laboratory and stored at -80°C until DNA extraction. 40 mL urine was used for the urinary microbiota analysis. Magnetic bead-based isolation of bacterial genomic DNA was carried out according to the manufacturer’s protocol with minor modifications (Beckman Coulter, United States) (Ling et al., 2017). The processes of DNA extraction and PCR set up were performed in a laminar air flow bench, illuminated with a UV lamp prior to use, in order to avoid possible contamination. Negative DNA extraction controls (containing lysis buffer and kit reagents only) were amplified and sequenced as the contamination controls. The amplicons were normalized, pooled, and sequenced on an Illumina MiSeq platform using a V3 reagent kit with 2 × 300 cycles. The 16S rRNA gene V3–V4 regions were amplified from the microbial genomic DNA (forward primer, 5′-ACTCCTACGGGAGGCAGCAG-3′; reverse primer, 5′-GGACTACHVGGGTWTCTAAT-3′). The 16S metagenomic sequencing data of the PreD group have also been used in a study (which is currently under review for publication) that compared the effect on urine microbiota of a transurethral catheterization sampling method to the effect of a new specially designed midstream urine collection technique.

### Bioinformatic and Statistical Analyses

The Quantitative Insights Into Microbial Ecology (QIIME, v1.9.0) pipeline was employed to process the sequencing data, as previously described (Caporaso et al., 2010). Raw sequencing reads with exact matches to the barcodes were assigned to respective samples and identified as valid sequences. The low-quality sequences were filtered out using the following criteria (Gill et al., 2006; Chen and Jiang, 2014): length < 150 bp, average Phred score < 20, contained ambiguous bases, and contained mononucleotide repeats > 8 bp. Paired-end reads were assembled using FLASH (Magoc and Salzberg, 2011). Operational taxonomic unit (OTU) picking using Vsearch v1.11.1, included dereplication(–derep_fulllength), cluster(–cluster_fast,–id 0.97), detectection of chimeras(–uchime_ref) (Rognes et al., 2016). The index of alpha diversity was calculated with QIIME based on a sequence similarity of 97%. Beta diversity was measured by unweighted UniFrac distance, which was also calculated by QIIME. The diversity and richness of the bacteria in the urine specimens were calculated using several estimates, such as OTUs, Chao1, and the Shannon and Simpson indices (which are measures of bacterial diversity). Principal coordinates analysis (PCoA) of weighted UniFrac distance was used to demonstrate the beta diversity. The taxonomic tree was visualized using GraPhlAn. Kruskal–Wallis rank sum tests and pairwise Wilcoxon tests were used to identify the different markers, and linear discriminant analysis (LDA) was used to score each feature in the LDA effect size (LEfSe) analysis. The output file was further analyzed using the Statistical Analysis of Metagenomic Profiles software package (version 2.1.3). To obtain insight into the possible functional pathways that differ between the PreD and PostD groups, PicRUst was used to calculate the contributions of various OTUs to known biological pathways using Kyoto Encyclopedia of Genes and Genomes (KEGG) databases (Langille et al., 2013; Vernocchi et al., 2017). KEGG pathways that were non-prokaryotic, had <2 sequences in each group, or had a difference in mean proportions < 0.1% were excluded from the analysis (Shoskes et al., 2016). White’s non-parametric *t*-test, using an alpha value of 0.05 with the Story false discovery rate (FDR)-controlling procedure, was used to control the FDR linked to multiple testing (Shoskes et al., 2016). Also, bacterial taxa with < 2 sequences in either group because they were suspected to contain contaminants, a relative abundance < 0.01% in either group, or a difference in mean proportions < 0.10% were removed from the analysis of the between-group differences in the relative abundance of bacterial taxa (Logares et al., 2014; Shoskes et al., 2016; Legione et al., 2018). All tests of significance were two-sided, and p/q < 0.05 was considered statistically significant.

The sequence data from this study were deposited in the GenBank Sequence Read Archive under accession number SRP 126710.

## Results

### Participant Characteristics

Thirty women undergoing cesarean section participated in the present study. Their characteristics are shown in **Table 1**. Their temperatures on the day PostD were slightly higher than PreD.

**Table 1:** Characteristics of participants (*n* = 30).

Parameter	Range and/or number of patients	Mean ± SD
Age (years)	19–43	31.43 ± 4.37
Gravidity (times)	1–5	2.57 ± 1.04
Parity (times)	1–2	1.80 ± 0.41
Temperature (°C) on the day of the first urine sample collection	36.30–37.30	36.80 ± 0.26
Temperature (°C) on the day after cesarean section	36.40–37.50	36.90 ± 0.31
Pulse (times/min)	72–100	85.00 ± 6.78
Respiration (times/min)	18–20	18.47 ± 0.57
Body mass index (kg/m^2^)	34.84–56.97	43.68 ± 4.72
Systolic blood pressure (mmHg)	93–138	114.80 ± 13.22
Diastolic blood pressure (mmHg)	50–88	71.00 ± 10.04
Fluid transfused (mL)	2200–4420	3635.87 ± 449.79

### Sequencing Results

The mean bacterial DNA concentration was 25.54 ng/μL for the 60 samples (the individual concentrations are shown in **Supplementary Table S1**). There were no bands on the gel after PCR amplification. When the negative control was sequenced, it yielded < 100 reads, and the sequences could not be assembled.

**FIGURE 1:**
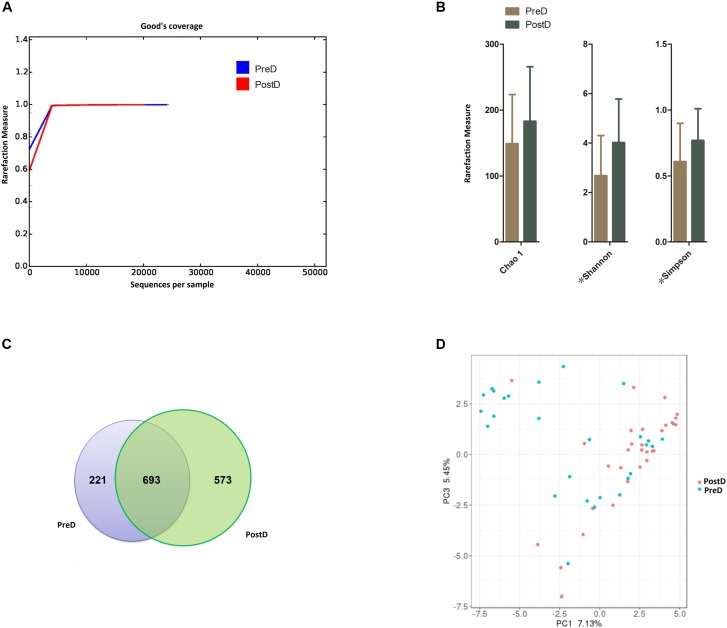
Bacterial diversity. **(A)** Good’s coverage was used to assess sequencing depth. **(B)** The Chao 1 index was used to measure richness, and the Shannon and Simpson rarefaction indices were applied to estimate diversity. ^∗^Indicates that a significant difference was found between groups (*p* < 0.05). **(C)** Venn diagram showing the number of OTUs shared between the PreD and PostD groups. **(D)** Principal coordinates analysis plot of the urinary microbiota based on the unweighted UniFrac metric. Blue and red dots represent the PostD and PreD groups, respectively. PreD means pre-delivery and PostD means post-delivery.

From the 60 samples, 2,367,239 raw sequences were produced, and 2,352,732 high-quality sequences remained after filtering out low-quality and short-length sequences, with a median read length of 420 bp (range, 391–528 bp). After filtering out the chimera, the mean number of sequences per barcoded sample used for downstream analysis was 32,972 reads. The total number of unique sequences from the PreD and PostD groups was 1,134,258, and they represented all phylotypes. Good’s coverage values in both the PreD and PostD groups were nearly 100% and the curves again level off, showing that the sequencing effort was sufficient to detect most of the OTUs (**Figure 1A**).

### The PostD Group Had Higher Bacterial Richness and Diversity Compared to the PreD Group

Chao 1, a bacterial richness index, was increased in the PostD group compared to the PreD group, but not significantly (**Figure 1B**) (*p* = 0.101). The Shannon and Simpson indices, measures of bacterial diversity, were significantly higher in the PostD group than the PreD group (*p* = 0.003; *p* = 0.020) (**Figure 1B**). The Venn diagram illustrated that 693 bacterial OTUs (accounting for 46.60%) were shared by the PreD and PostD groups (**Figure 1C**). The PCoA illustrated that the OTUs in the PreD and PostD groups could not completely separated (**Figure 1D**).

### The PostD Group Had More Detectable Bacteria at All Taxon Levels, With Some Bacteria Showing Differences Between Groups

When the bacterial phyla were analyzed, 21 bacterial phyla were detected and all of them were detectable in the PostD group, while 6 were non-detectable in the PreD group, including Gemmatimonadetes and Planctomycetes. Although Firmicutes, Actinobacteria, Proteobacteria, Bacteroidetes, and Thermi were the most abundant bacteria in both the PreD and PostD groups, Firmicutes was significantly enriched in the PreD group compared to the PostD group (*q* = 0.000). In addition, the relative abundance of Proteobacteria was significantly decreased in the PreD group compared to the PostD group (*q* = 0.000) (**Supplementary Table S2**).

**FIGURE 2:**
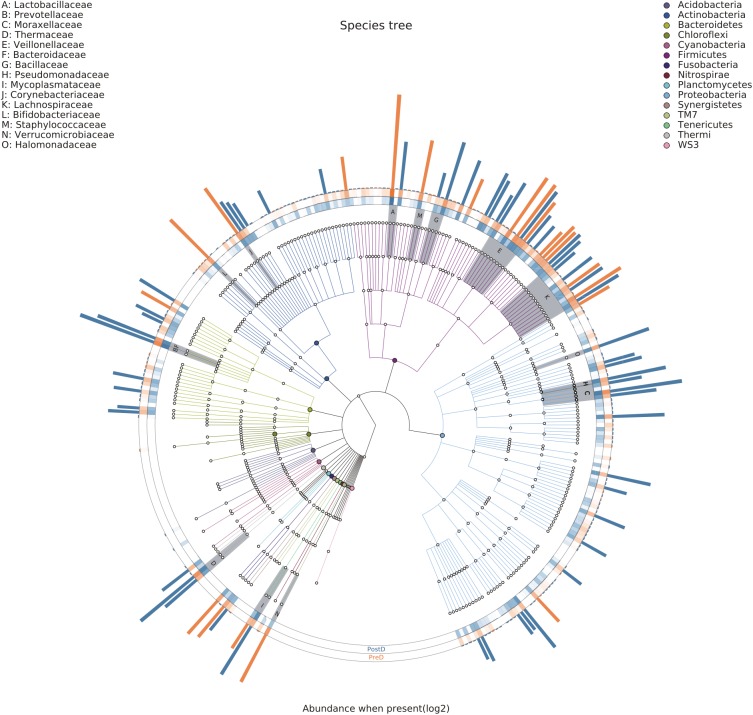
Taxonomic visualization. Taxonomic tree visualized using GraPhlAn. The color of each node is consistent with the color of the corresponding bacterial phylum node located in the upper left corner of the graph. Each letter in the inner circle is consistent with the letter located in the upper right corner of the graph. The orange and blue columns around the circle represent the relative abundances of bacterial families in the PreD and PostD groups, respectively. PreD means pre-delivery and PostD means post-delivery.

At the family level, 135 and 116 bacteria families were present in the PostD and PreD groups, respectively. The bacterial families present in the PreD and PostD groups are shown in **Supplementary Table S3**. Bifidobacteriaceae, Lactobacillaceae, Lachnospiraceae, Thermaceae, and Prevotellaceae were the most abundant bacterial families in the present study (**Supplementary Figure S1**). The PreD group had higher levels of Lactobacillaceae and Coriobacteriaceae than the PostD group (*q* = 0.003; *q* = 0.044), while the PostD group had more abundant Bacteroidaceae, Comamonadaceae, Halomonadaceae, Methylobacteriaceae, and Micrococcaceae than the PreD group (*q* = 0.020; *q* = 0.016; *q* = 0.031; *p* = 0.007; *p* = 0.023) (**Supplementary Figure S2**). Bacterial families belonging to the phylum Proteobacteria were mainly found in the PostD group, such as Pseudomonadaceae, Halomonadaceae, and Moraxellaceae. Similarly, bacterial families belonging to the phylum Bacteroidetes were also mainly found in the PostD group. Bacterial families in the phylum Firmicutes were frequently detected; half of them were found in the PreD group, including Lactobacillaceae, Staphylococcaceae, and Veillonellaceae (**Figure 2**).

**FIGURE 3:**
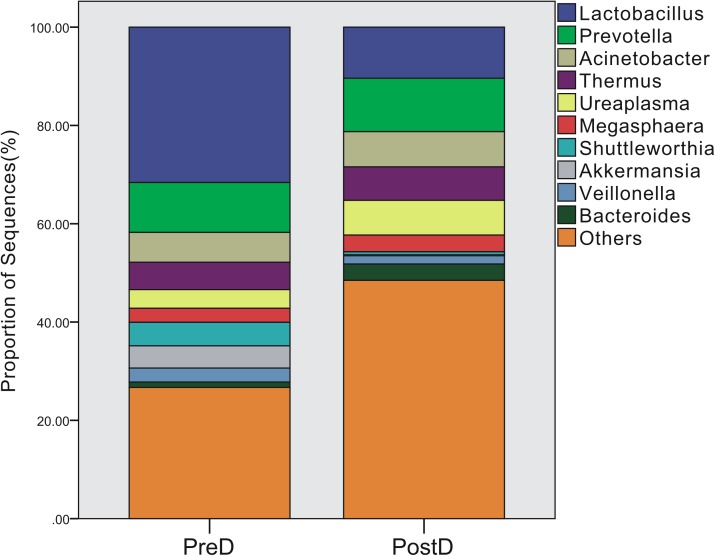
Bacterial genera profile. Top 10 most abundant bacterial genera in the PreD and PostD groups. PreD means pre-delivery and PostD means post-delivery.

**FIGURE 4:**
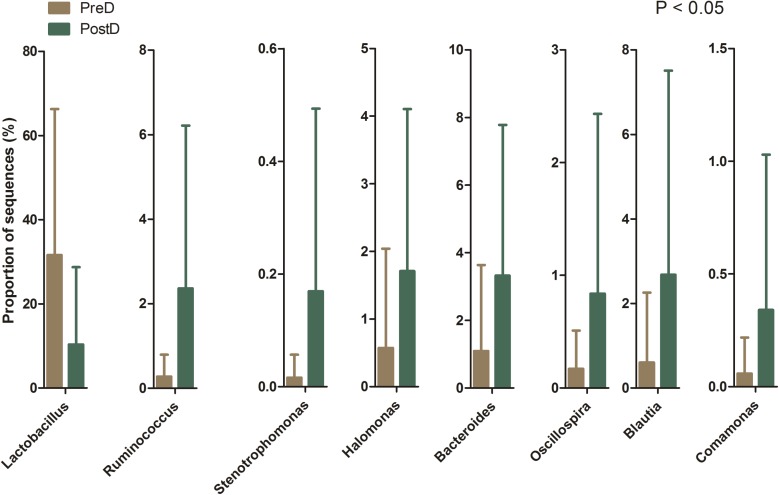
Bacterial genera differences. Bacterial genera showing significant differences in relative abundance between the PreD and PostD groups. PreD means pre-delivery and PostD means post-delivery.

A total of 219 bacterial genera were found in the present study, and 181 genera were detected in the PreD group, while 209 genera were noted in the PostD group. The most abundant bacterial genus in the PreD group was *Lactobacillus* (accounting for 31.61%), while *Prevotella* was ranked as the most abundant bacteria in the PostD group (accounting for 10.88%). The top 10 most abundant bacteria in the PreD and PostD groups are shown in **Figure 3**. *Lactobacillus* was significantly lower in the PostD group compared to the PreD group, whereas *Ruminococcus* and *Bacteroides* were significantly increased in the PostD group compared to the PreD group (**Figure 4**).

### Biomarkers Associated With Cesarean Section

The LEfSe analysis demonstrated that Bacilli, Lactobacillaceae, *Lactobacillus*, Lactobacillales, Firmicutes, Mobiluncus, and Actinomycetaceae could differentiate PreD specimens from the PostD specimens (**Supplementary Figure S3**).

### Metabolic Pathways Were Correlated With Cesarean Delivery

**FIGURE 5:**
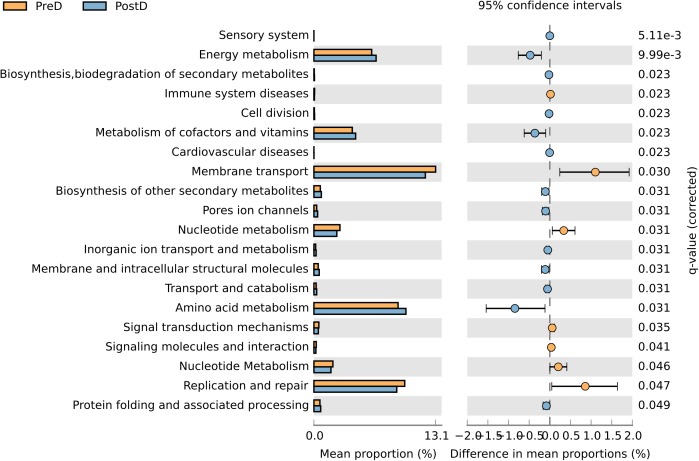
Contributions of OTUs to known biological metabolic pathways. Functional analysis of urinary microbiota from the PreD and PostD groups. Microbial pathways predicted to be differentially regulated based on microbiome differences between the PreD (orange) and PostD (blue) groups. Upregulated pathways are represented by a higher mean proportion of expression. *q*-values are based on White’s nonparametric *t*-test and corrected using the Story false discovery rate-controlling procedure.

Twenty metabolic pathways were associated with the PreD and PostD groups. Energy metabolism, metabolism of cofactors and vitamins, and amino acid metabolism were strengthened in the PostD group compared to the PreD group. However, membrane transport, nucleotide metabolism, and replication and repair lessened in the PostD group (**Figure 5**).

## Discussion

The associations between the human microbiota and health status have been of great concern to researchers and clinicians in recent decades. Although some studies have demonstrated that delivery mode impacted on the human microbiota, the focus of these studies was on the infant gut microbiota (Dominguez-Bello et al., 2010; Jakobsson et al., 2014; Liu et al., 2015; Chu et al., 2017), and few focused on the effects of delivery mode on the maternal microbiota. The bladder, previously considered sterile, has been demonstrated to have a unique bacterial community and the urinary microbiota has been found to be associated with diseases including urologic disorders, cancer, burn injuries, sexually transmitted infection, metabolic disease, bacterial vaginosis, etc. (Nelson et al., 2010; Aragon et al., 2016; Gottschick et al., 2017; Ling et al., 2017; Plichta et al., 2017; Shrestha et al., 2018). As previous studies reported that postpartum complications such as UTI are associated with the mode of delivery (Leth et al., 2009; Hung et al., 2016), the present study explored whether the characteristics of the urinary microbiota are associated with cesarean section and postpartum health status.

The present study indicates that there is a greater microbial diversity and a greater bacterial relative abundance at all taxonomic levels (including phylum, family, and genus) in the PostD group compared to the PreD group. This shows that cesarean section, which consists of surgery and delivery, has a profound influence on women’s urinary microbial composition. Interestingly, urinary microbial diversity has also been found to be significantly increased in burn patients compared to controls (Plichta et al., 2017). This phenomenon is also similar to that identified in a study by Howard et al. (2017) which found that significant changes in gut microbial diversity occurred early after severe injury. Cesarean section is a traumatic experience for women (Ryding et al., 1997), and the above phenomena suggest that microbial diversity increases after trauma such as cesarean section. The PCoA indicated that cesarean section was not the only influencing factor responsible for the urinary microbiota changes in women undergoing cesarean section. As demonstrated by studies of the human gut and vaginal microbiota, the sharp decline in estrogen and progesterone levels after delivery may also affect the urinary microbiota (Koren et al., 2012; MacIntyre et al., 2015; Neuman et al., 2015). Therefore, the alteration of urinary microbiota caused by delivery via cesarean section may not only be associated with the cesarean section, but also with changes in hormone levels resulting from delivery (which can also occur after vaginal delivery). The number of OTUs shared between the PreD and PostD groups was lower than the number of OTUs detected overall, illustrating that cesarean section delivery played an important role in the dysbiosis of the urinary microbiota in women. Clinicians may need to be aware of possible cesarean section delivery-related complications associated with the imbalance of urinary microbiota.

Gemmatimonadetes and Planctomycetes were not found in the PreD group, while they were found in the PostD group. In addition, Cyanobacteria, Chloroflexi, and Acidobacteria showed a great shift after delivery. Little is known about these bacteria and further research should be carried out to assess their roles in the urinary microbiota and the possible connection to delivery modes.

Some genera belonging to the phylum Firmicutes such as *Lactobacillus* sharply decreased in the PostD group, which is similar to the change in *Lactobacillus* shown in a previous study by Plichta et al. (2017) on the urinary microbiota in burn patients. In their study, the relative abundance of *Lactobacillus* continuously decreased after burn injury. Both cesarean section and burn injuries can lead to post-traumatic stress disorder (Lopez et al., 2017; Plichta et al., 2017). Post-traumatic stress disorder occurs more frequently in women following cesarean section than in women with normal delivery (Lopez et al., 2017). The elevated stress disorder can predict reduced estrogen level in women (Roney and Simmons, 2015). The postpartum period is associated with a significant decrease in estrogen (Nuriel-Ohayon et al., 2016), and the level of estrogen has been demonstrated to be responsible for the proportion of *Lactobacillus* in the bladder (Karstens et al., 2016). Post-delivery disturbances in the vaginal bacterial community, in terms of a decreased proportion of *Lactobacillus*, have also been demonstrated to occur. A study by Digiulio et al. found that *Lactobacillus* significantly decreased and was replaced by *Peptoniphilus*, *Prevotella*, and *Anaerocuccus* after delivery (DiGiulio et al., 2015). These findings suggest that hormone alterations caused by delivery play an important role in regulating the microbiota, including the urinary microbiota and vaginal microbiota, after delivery (DiGiulio et al., 2015; Nuriel-Ohayon et al., 2016). Therefore, the alteration of urinary microbiota is not only attributed to cesarean section, but also to the hormonal changes associated with the cesarean section delivery mode. Proteobacteria increased in the PostD group compared to the PreD group and detectable bacterial families in the phylum Proteobacteria were mainly found in the PostD group, suggesting that it may play a role in postpartum diseases including infections. Emerging evidence has demonstrated that increases in the expression of genes associated with inflammatory disease coincide with expansion of Proteobacteria blooms (Miyasaka et al., 2013; Mizuta et al., 2016; Nagao-Kitamoto et al., 2016). For example, the genus *Pseudomonas* (which belongs to the phylum Proteobacteria) has been frequently found to be correlated with infections (Micek et al., 2005; Pobiega et al., 2016; Boutin et al., 2017). In our study, the bacterial order Pseudomonadales and genus *Pseudomonas* (which both belong to the phylum Proteobacteria) were distinguishing markers for PostD specimens. Several recent studies reported that Proteobacteria was frequently detected in subjects with asymptomatic sexually transmitted infection (Nelson et al., 2010), urgency urinary incontinence patients (Karstens et al., 2016), and UTI patients (Willner et al., 2014; Santiago-Rodriguez et al., 2015), indicating that Proteobacteria might play a role in complications in postpartum women. Yoo et al. (2016) reported that women who had undergone delivery had a lower abundance of *Pseudomonas* than non-pregnant women. We found that *Pseudomonas* was enriched in the PostD specimens; similar results were reported in a study by Toscano et al., 2017 who compared the vaginal microbiota between women with cesarean section and those who underwent vaginal delivery: women who underwent cesarean section had a higher abundance of *Pseudomonas* than women who delivered vaginally. The present results as well as those of Toscano et al. (2017) suggest that low levels of *Pseudomonas* may play a role in maintaining the vaginal health and normal urinary functions of pregnant women, and that cesarean section might disrupt this balance.

Although 22 bacterial families were not found in the PreD group, they were detectable in the PostD group. Their function in the human urinary microbiota is unclear, as almost all of them have not previously been reported. Interestingly, Bacteroidaceae increased after cesarean section, which was similar to another urinary microbiota study on chronic prostatitis/chronic pelvic pain syndrome patients (Shoskes et al., 2016). In their study, Shoskes et al. (2016) reported that the urinary microbiota of patients with gastrointestinal symptoms was dominated by Bacteroidaceae (Shoskes et al., 2016). This suggests that Bacteroidaceae in the urinary microbiota is not indicative of health.

Ollberding et al. (2016) reported that the relative abundance of *Ruminococcus* was higher in women who underwent preterm delivery than normal delivery controls. In addition, Ling et al. (2017) noted that *Ruminococcus* could explain the presence of interleukin-8 in urine. Hence, the increased level of *Ruminococcus* spp. in our present study may be correlated with complications after cesarean section.

After delivery, the woman subjects initiated lactation. During the lactation stage, basal metabolic rates and mobilization of fat stores increase (Gunderson, 2014), which might be responsible for the strengthened energy metabolism in the PostD group. Interestingly, although women who underwent cesarean section would theoretically have had many complications, none the subjects in our present study had postpartum complications (except for back pain). This might be due to the higher proportion of pathways related to the metabolism of cofactors and vitamins resulting from the urinary microbiota alterations in the PostD group, as some vitamins (such as vitamins A, B, and C) play roles in boosting the immune system (Arigony et al., 2013). Notably, the pathway related to membrane transport proteins was reduced in the present study. This suggests that clinicians should consider taking measures to restore the membrane transport proteins, as they play important roles in cellular functions (Mishra et al., 2014), which are correlated with women’s recovery after delivery (Groer et al., 2015). In addition, the pathway related to replication and repair was impaired after delivery, which suggests that tissue injury caused by cesarean section is correlated with urinary microbiota alteration, indicating that measures for balancing urinary microbiota dysbiosis may play a role in the recovery of postpartum women.

### Limitations

The main limitation in our present study is the lack of control group. Therefore, it is hard to rule out the influences on urinary microbiota caused by hormonal changes correlated with delivery. We could not perform a case control study as it was impossible to collect urine specimens from pregnant women who did not need a cesarean section, because transurethral catheterization is an invasive procedure and it is unnecessary for women undergoing normal delivery. Importantly, performing transurethral catheterization in this population does not meet our ethical standards. Although midstream urine specimens are thought to be representative of the microbiota in the bladder, they are likely to contain traces of contaminating bacteria from the genital tract (Ainsworth, 2017), so we could not use midstream urine specimens. Thus, we could not use a case control study approach in the present study. There is an urgent need to design a non-invasive urine specimen collection technique in the future, which would be useful for comparing urinary microbiota alterations caused by various surgeries such as cesarean section, as the alterations might provide hints for preventing complications after surgery.

Another limitation is that the present study could not rule out the potential effects of anesthesia, which has been demonstrated to increase the abundance of *Ruminococcus* in horse fecal microbiota, 48 h after anesthesia (Schoster et al., 2016). Firstly, this limitation arose due to the inability to recruit women who had elected to receive epidural analgesia during labor, owing to widespread belief (among both pregnant women and health care providers in China) that epidural analgesia is harmful to the mother and baby (Hu et al., 2016). Secondly, catheterization was refused, even among women who elected to receive epidural analgesia. Thirdly, women exhibit high levels of lochia (vaginal discharge following delivery). It is difficult to obtain urine samples in which lochia is absent, as participants refused to undergo sample collection procedures owing to the discomfort involved. Finally, it is difficult to disinfect the perineum area because the flow of lochia; as a result, the collection of uncontaminated urine samples was difficult to achieve.

In addition, our present study only explored the urinary microbiota changes caused by cesarean section. Future work needs to be focused on animal models to explain why cesarean section leads to the alterations, which might be helpful to guide clinicians to take more specific measures to prevent urinary microbiota-related disease.

## Conclusion

To our knowledge, this is the first study investigating the alterations in bladder microbiota caused by cesarean section. Firstly, women’s urinary microbiota is significantly altered before and after delivery. It is valuable to note that pathogenic bacteria dramatically increased in the PostD group compared to the PreD group; therefore, clinicians should consider therapy for women who have given birth to depress the growth of bladder pathogens, which might be helpful to prevent postpartum complications such as infections. Secondly, the changes in the metabolic pathways reflected the composition of the urinary microbiota, which might be useful information for clinicians investigating the etiological factors of metabolic disease in postpartum women. Future work using a larger sample size is needed to confirm this result and future work is needed to explore how the alteration of urinary microbiota leads to changes in metabolic pathways in postpartum women. Finally, alterations in the PostD group may be responsible for postpartum symptoms including back pain. Thus, regulation of the urinary microbiota is a potential new therapy to control back pain in postpartum women.

## Author Contributions

YC and WW conceived and designed the study. FL generated the sequencing data. FL and LL collected the samples. HJ and RY conducted the urine culturing. SD and FL extracted the bacterial DNA. LL, LC, and FL analyzed the data. FL carried out the computational analysis. YC interpreted the data. FL and YC drafted the manuscript.

## Conflict of Interest Statement

The authors declare that the research was conducted in the absence of any commercial or financial relationships that could be construed as a potential conflict of interest.

## Supplementary Material

FIGURE S1 Bacterial family profile. Top 10 most abundant bacterial families in the PreD and PostD groups. PreD means pre-delivery and PostD means post-delivery.

FIGURE S2 Bacterial family differences. Bacterial genera showing significant differences in relative abundance between the PreD and PostD groups. PreD means pre-delivery and PostD means post-delivery.

FIGURE S3 Cladogram of differentially abundant microbiota taxa. **(A)** LEfSe cladogram showing the most differentially abundant taxa between the PreD and PostD groups. Taxonomic cladogram obtained from LEfSe analysis of 16S rRNA sequences. Blue and red represents taxa enriched in the PreD and PostD groups, respectively. The brightness of each dot is proportional to its effect size. **(B)** Only taxa meeting an LDA threshold > 2.0 are shown. PreD means pre-delivery and PostD means post-delivery.

TABLE S1 Relative abundance of bacterial phyla in the PreD and PostD groups.

TABLE S2 Presence of bacterial families.
