# Supplementary figures and images for: Alterations in the Urinary Microbiota Are Associated With Cesarean Delivery
Source: Front Microbiol. 2018 Sep 12;9:2193. doi: 10.3389/fmicb.2018.02193 (PMC6143726; doi:10.3389/fmicb.2018.02193)

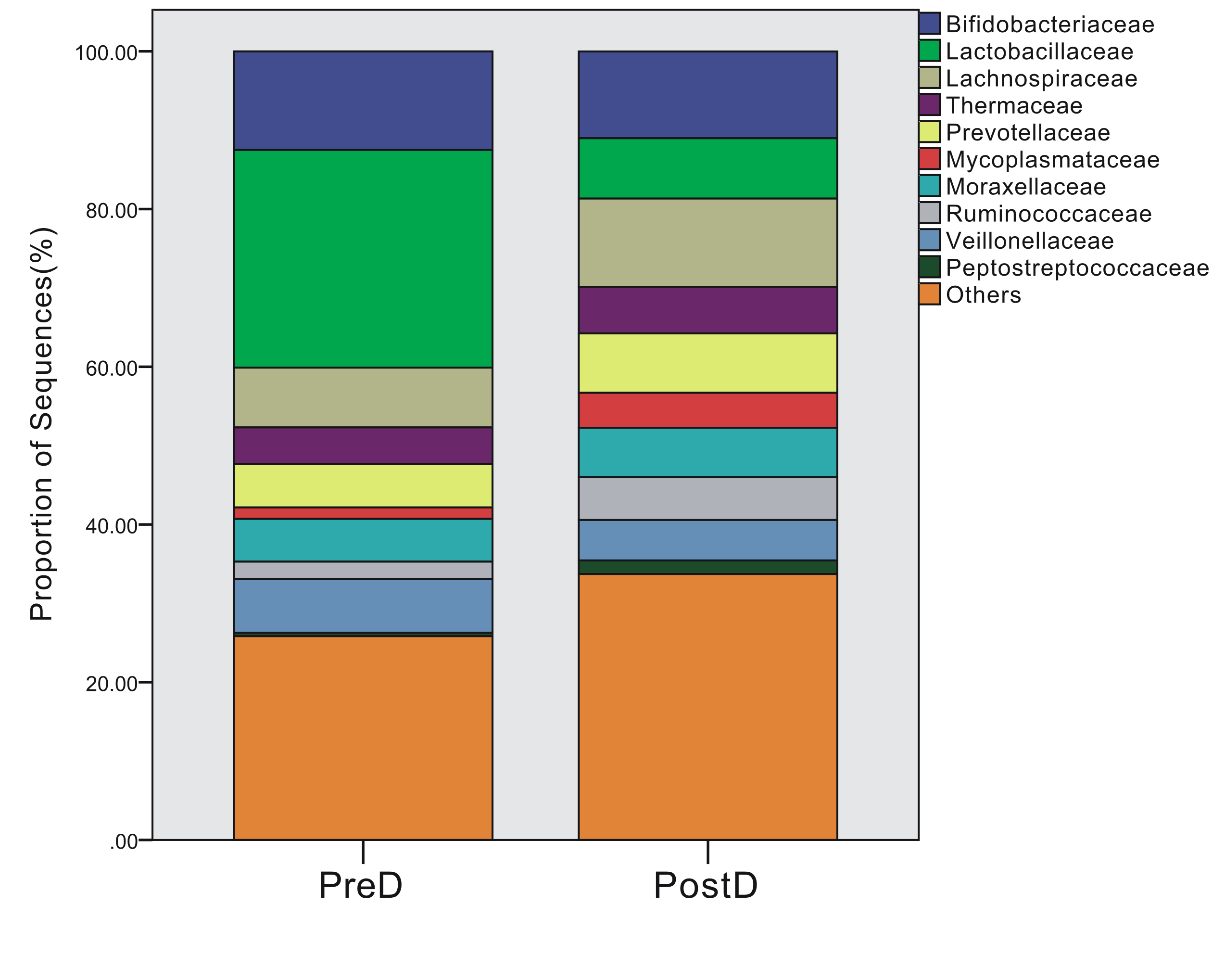

Supplement: FIGURE S1 — Bacterial family profile. Top 10 most abundant bacterial families in the PreD and PostD groups. PreD means pre-delivery and PostD means post-delivery. [file Image_1.TIF]

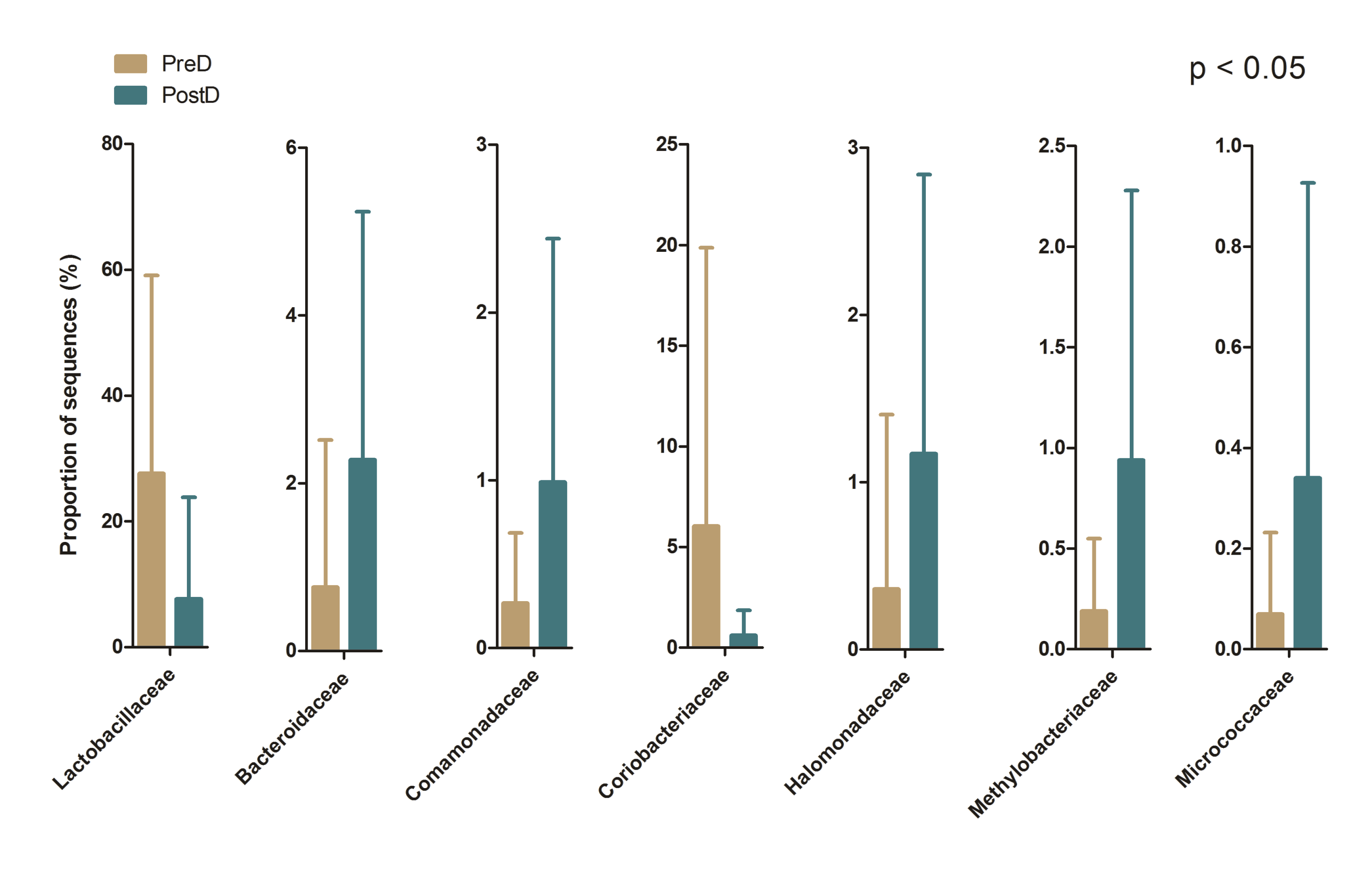

Supplement: FIGURE S2 — Bacterial family differences. Bacterial genera showing significant differences in relative abundance between the PreD and PostD groups. PreD means pre-delivery and PostD means post-delivery. [file Image_2.TIF]

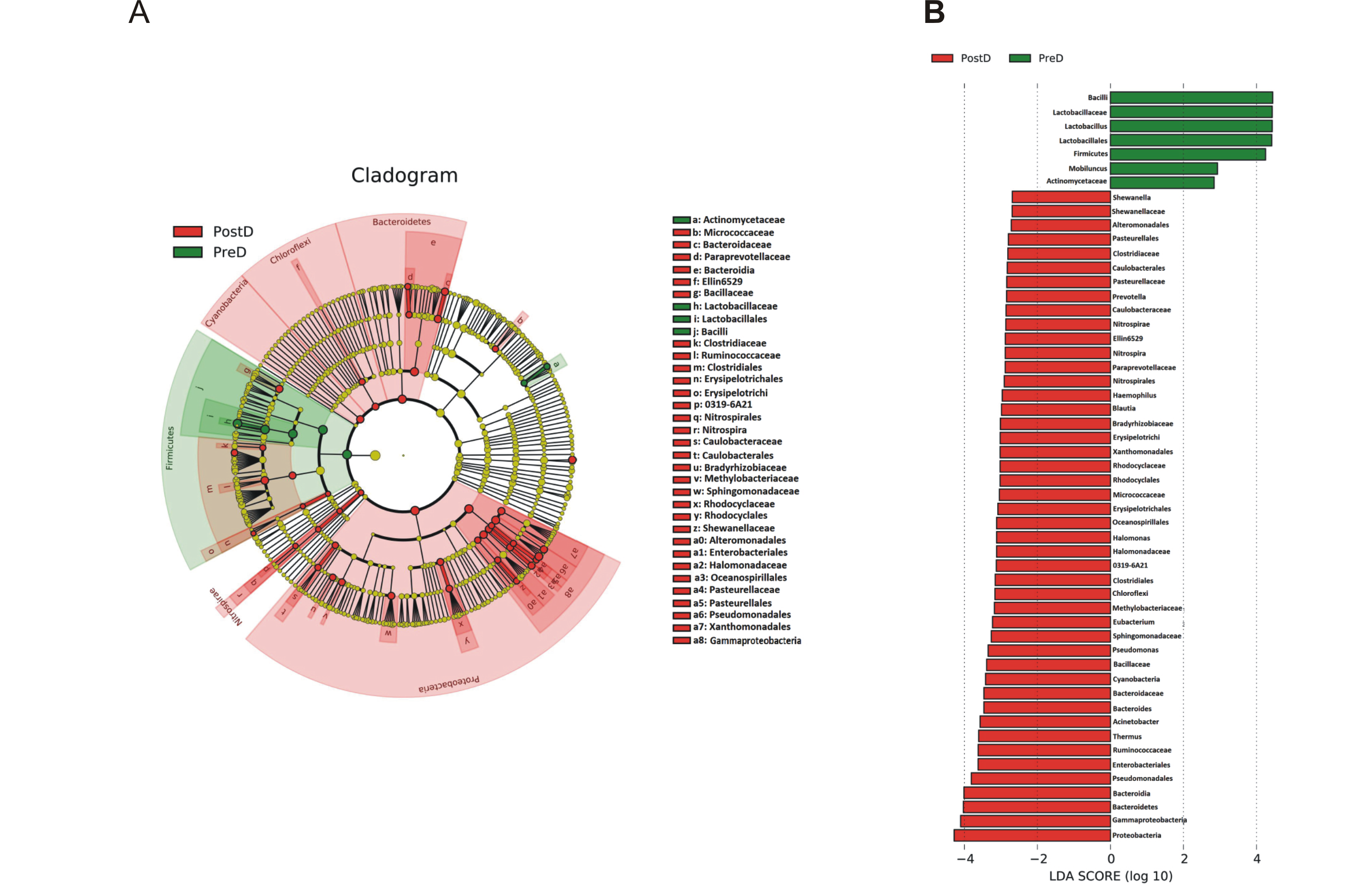

Supplement: FIGURE S3 — Cladogram of differentially abundant microbiota taxa. (A) LEfSe cladogram showing the most differentially abundant taxa between the PreD and PostD groups. Taxonomic cladogram obtained from LEfSe analysis of 16S rRNA sequences. Blue and red represents taxa enriched in the PreD and PostD groups, respectively. The brightness of each dot is proportional to its effect size. (B) Only taxa meeting an LDA threshold > 2.0 are shown. PreD means pre-delivery and PostD means post-delivery. [file Image_3.TIF]
