# Supplementary material for: Alterations in the Urinary Microbiota Are Associated With Cesarean Delivery
Source: Front Microbiol. 2018 Sep 12;9:2193. doi: 10.3389/fmicb.2018.02193 (PMC6143726; doi:10.3389/fmicb.2018.02193)
Supplement: TABLE S1 — Relative abundance of bacterial phyla in the PreD and PostD groups. [file Table_1.DOCX]

**Table S1 Bacterial DNA concentration**

| **Sample ID** | **Concentration (ng/µl)** | **OD (260/280)** | **OD (260/230)** |
| --- | --- | --- | --- |
| PreD1 | 9.92 | 2.07 | 1.19 |
| PreD2 | 7.08 | 1.42 | 0.99 |
| PreD3 | 2.52 | 1.75 | 0.78 |
| PreD4 | 5.15 | 1.33 | 0.89 |
| PreD5 | 4.86 | 1.41 | 1.14 |
| PreD6 | 4.39 | 1.35 | 0.98 |
| PreD7 | 5.47 | 1.33 | 0.65 |
| PreD8 | 26.93 | 1.54 | 1.29 |
| PreD9 | 4.95 | 1.16 | 1.07 |
| PreD10 | 77.17 | 1.75 | 1.57 |
| PreD11 | 13.94 | 1.50 | 0.85 |
| PreD12 | 0.01 | 1.11 | 0.45 |
| PreD13 | 3.92 | 1.04 | 0.85 |
| PreD14 | 9.44 | 1.44 | 1.66 |
| PreD15 | 4.35 | 0.52 | 0.39 |
| PreD16 | 431.79 | 0.83 | 0.72 |
| PreD17 | 131.31 | 1.69 | 1.60 |
| PreD18 | 5.51 | 1.78 | 1.43 |
| PreD19 | 4.37 | 2.84 | 1.33 |
| PreD20 | 13.74 | 1.75 | 1.31 |
| PreD21 | 6.22 | 2.39 | 0.92 |
| PreD22 | 2.42 | 1.85 | 0.61 |
| PreD23 | 0.01 | 2.72 | 0.47 |
| PreD24 | 78.1 | 1.82 | 2.06 |
| PreD25 | 18.69 | 1.67 | 1.22 |
| PreD26 | 4.51 | 1.46 | 0.34 |
| PreD27 | 2.61 | 2.12 | 0.78 |
| PreD28 | 21.41 | 1.76 | 2.66 |
| PreD29 | 16.47 | 1.67 | 4.36 |
| PreD30 | 22.74 | 1.61 | 1.74 |
| PostD1 | 4.20 | 1.45 | 1.19 |
| PostD2 | 2.37 | 1.08 | 1.23 |
| PostD3 | 2.08 | 0.34 | 0.56 |
| PostD4 | 91.23 | 1.30 | 0.49 |
| PostD5 | 50.12 | 0.76 | 0.51 |
| PostD6 | 3.42 | 1.16 | 0.31 |
| PostD7 | 0.01 | 2.75 | 0.36 |
| PostD8 | 2.67 | 1.28 | 0.55 |
| PostD9 | 0.01 | 0.63 | 0.30 |
| PostD10 | 8.90 | 1.63 | 0.52 |
| PostD11 | 188.36 | 1.58 | 0.78 |
| PostD12 | 55.02 | 1.59 | 0.92 |
| PostD13 | 3.38 | 1.20 | 0.57 |
| PostD14 | 3.63 | 1.39 | 0.42 |
| PostD15 | 2.19 | 2.36 | 0.46 |
| PostD16 | 13.35 | 1.01 | 0.74 |
| PostD17 | 24.05 | 0.62 | 0.48 |
| PostD18 | 1.79 | 3.80 | 0.78 |
| PostD19 | 2.14 | 1.42 | 0.53 |
| PostD20 | 0.01 | 1.15 | 0.53 |
| PostD21 | 3.62 | 1.50 | 0.84 |
| PostD22 | 0.01 | 3.75 | 0.46 |
| PostD23 | 2.32 | 2.67 | 0.41 |
| PostD24 | 11.67 | 1.70 | 1.18 |
| PostD25 | 0.01 | 1.45 | 0.42 |
| PostD26 | 1.83 | 1.10 | 0.85 |
| PostD27 | 5.37 | 1.19 | 0.88 |
| PostD28 | 18.65 | 1.52 | 3.38 |
| PostD29 | 24.79 | 1.60 | 2.28 |
| PostD30 | 65.76 | 1.84 | 1.02 |
