# Supplementary material for: Alterations in the Urinary Microbiota Are Associated With Cesarean Delivery
Source: Front Microbiol. 2018 Sep 12;9:2193. doi: 10.3389/fmicb.2018.02193 (PMC6143726; doi:10.3389/fmicb.2018.02193)
Supplement: TABLE S2 — Presence of bacterial families. [file Table_2.DOCX]

**Table S2 Relative abundance of bacterial phyla in the two groups**

| **Taxon** | **PreD** | **PostD** | **q-value** |
| --- | --- | --- | --- |
| Cyanobacteria | 0.17±0.41 | 2.56±10.78 | 0.000 |
| Chloroflexi | 0.19±0.63 | 1.84±4.20 | 0.000 |
| Firmicutes | 48.88±29.75 | 33.95±20.10 | 0.000 |
| Acidobacteria | 0.21±0.59 | 1.47±3.54 | 0.000 |
| Proteobacteria | 11.49±19.42 | 21.75±21.31 | 0.000 |
