# Supplementary material for: Alterations in the Urinary Microbiota Are Associated With Cesarean Delivery
Source: Front Microbiol. 2018 Sep 12;9:2193. doi: 10.3389/fmicb.2018.02193 (PMC6143726; doi:10.3389/fmicb.2018.02193)
Supplement: Supplementary file 6 [file Table_3.DOCX]

**Table S3 Presence of bacterial families**

| **PreD and PostD** | **PreD** | **PostD** |
| --- | --- | --- |
| Lactobacillaceae | Lactobacillaceae | Lachnospiraceae |
| Bifidobacteriaceae | Bifidobacteriaceae | Bifidobacteriaceae |
| Lachnospiraceae | Lachnospiraceae | Lactobacillaceae |
| Veillonellaceae | Veillonellaceae | Prevotellaceae |
| Coriobacteriaceae | Coriobacteriaceae | Moraxellaceae |
| Prevotellaceae | Prevotellaceae | Thermaceae |
| Moraxellaceae | Moraxellaceae | Ruminococcaceae |
| Thermaceae | Thermaceae | Veillonellaceae |
| Verrucomicrobiaceae | Verrucomicrobiaceae | Mycoplasmataceae |
| Ruminococcaceae | Ruminococcaceae | Xanthomonadaceae |
| Xanthomonadaceae | Xanthomonadaceae | Bacteroidaceae |
| Mycoplasmataceae | Mycoplasmataceae | Enterobacteriaceae |
| Corynebacteriaceae | Corynebacteriaceae | Peptostreptococcaceae |
| Bacillaceae | Bacillaceae | Pseudomonadaceae |
| Staphylococcaceae | Staphylococcaceae | Sphingomonadaceae |
| Tissierellaceae | Tissierellaceae | S24-7 |
| Bacteroidaceae | Bacteroidaceae | Bacillaceae |
| Campylobacteraceae | Campylobacteraceae | Halomonadaceae |
| Streptococcaceae | Streptococcaceae | Rhodocyclaceae |
| Actinomycetaceae | Actinomycetaceae | Comamonadaceae |
| Peptostreptococcaceae | Peptostreptococcaceae | Caulobacteraceae |
| Caulobacteraceae | Caulobacteraceae | Tissierellaceae |
| Enterobacteriaceae | Enterobacteriaceae | Methylobacteriaceae |
| Halomonadaceae | Halomonadaceae | Corynebacteriaceae |
| Rhodocyclaceae | Rhodocyclaceae | Streptococcaceae |
| Fusobacteriaceae | Fusobacteriaceae | Microbacteriaceae |
| Deinococcaceae | Deinococcaceae | Coriobacteriaceae |
| Mycobacteriaceae | Mycobacteriaceae | Sinobacteraceae |
| Sinobacteraceae | Sinobacteraceae | Bradyrhizobiaceae |
| Leptotrichiaceae | Leptotrichiaceae | Porphyromonadaceae |
| Comamonadaceae | Comamonadaceae | Mycobacteriaceae |
| Paenibacillaceae | Paenibacillaceae | Paenibacillaceae |
| Pseudomonadaceae | Pseudomonadaceae | Staphylococcaceae |
| Porphyromonadaceae | Porphyromonadaceae | Clostridiaceae |
| Alcaligenaceae | Alcaligenaceae | Enterococcaceae |
| Sphingomonadaceae | Sphingomonadaceae | Micrococcaceae |
| Methylobacteriaceae | Methylobacteriaceae | Weeksellaceae |
| Weeksellaceae | Weeksellaceae | Alcaligenaceae |
| Clostridiaceae | Clostridiaceae | Sphingobacteriaceae |
| Rhodospirillaceae | Rhodospirillaceae | Propionibacteriaceae |
| Sphingobacteriaceae | Sphingobacteriaceae | mb2424 |
| S24-7 | S24-7 | Paraprevotellaceae |
| Bacteriovoracaceae | Bacteriovoracaceae | Deinococcaceae |
| Hyphomicrobiaceae | Hyphomicrobiaceae | Kouleothrixaceae |
| Brucellaceae | Brucellaceae | Pasteurellaceae |
| Intrasporangiaceae | Intrasporangiaceae | Leptotrichiaceae |
| Micrococcaceae | Micrococcaceae | Brucellaceae |
| Odoribacteraceae | Odoribacteraceae | Flavobacteriaceae |
| Paraprevotellaceae | Paraprevotellaceae | Dethiosulfovibrionaceae |
| Cytophagaceae | Cytophagaceae | Shewanellaceae |
| Shewanellaceae | Shewanellaceae | mitochondria |
| Dethiosulfovibrionaceae | Dethiosulfovibrionaceae | 0319-6A21 |
| Bradyrhizobiaceae | Bradyrhizobiaceae | Campylobacteraceae |
| Nitrospiraceae | Nitrospiraceae | Rhodospirillaceae |
| Helicobacteraceae | Helicobacteraceae | Solirubrobacteraceae |
| Propionibacteriaceae | Propionibacteriaceae | Streptosporangiaceae |
| Geodermatophilaceae | Geodermatophilaceae | Gaiellaceae |
| Desulfovibrionaceae | Desulfovibrionaceae | Verrucomicrobiaceae |
| C111 | C111 | Nitrospiraceae |
| Microbacteriaceae | Microbacteriaceae | Fusobacteriaceae |
| Brevibacteriaceae | Brevibacteriaceae | Actinomycetaceae |
| Neisseriaceae | Neisseriaceae | Helicobacteraceae |
| Rhodobacteraceae | Rhodobacteraceae | Nocardioidaceae |
| Rhizobiaceae | Rhizobiaceae | Koribacteraceae |
| Acetobacteraceae | Acetobacteraceae | Hyphomicrobiaceae |
| mb2424 | mb2424 | Neisseriaceae |
| Aerococcaceae | Aerococcaceae | Rhodobacteraceae |
| Beijerinckiaceae | Beijerinckiaceae | Odoribacteraceae |
| Turicibacteraceae | Turicibacteraceae | Dermabacteraceae |
| Nocardioidaceae | Nocardioidaceae | Rhizobiaceae |
| Rs-045 | Rs-045 | A4b |
| Kouleothrixaceae | Kouleothrixaceae | Oxalobacteraceae |
| Enterococcaceae | Enterococcaceae | Methylocystaceae |
| Ellin6075 | Ellin6075 | Aeromonadaceae |
| Gaiellaceae | Gaiellaceae | Desulfovibrionaceae |
| Flavobacteriaceae | Flavobacteriaceae | Christensenellaceae |
| Aeromonadaceae | Aeromonadaceae | Rikenellaceae |
| Pasteurellaceae | Pasteurellaceae | PRR-10 |
| Nitrosomonadaceae | Nitrosomonadaceae | Pseudonocardiaceae |
| Dermabacteraceae | Dermabacteraceae | Ellin6075 |
| Xenococcaceae | Xenococcaceae | Brevibacteriaceae |
| Chitinophagaceae | Chitinophagaceae | Xenococcaceae |
| Oxalobacteraceae | Oxalobacteraceae | Beijerinckiaceae |
| Planococcaceae | Planococcaceae | Turicibacteraceae |
| Hyphomonadaceae | Hyphomonadaceae | Chitinophagaceae |
| Bdellovibrionaceae | Bdellovibrionaceae | Cytophagaceae |
| Erysipelotrichaceae | Erysipelotrichaceae | Erysipelotrichaceae |
| Methylocystaceae | Methylocystaceae | Bdellovibrionaceae |
| Gemellaceae | Gemellaceae | Gemellaceae |
| Williamsiaceae | Williamsiaceae | C111 |
| Alteromonadaceae | Alteromonadaceae | Geobacteraceae |
| ACK-M1 | ACK-M1 | ACK-M1 |
| Burkholderiaceae | Burkholderiaceae | Acetobacteraceae |
| Dermacoccaceae | Dermacoccaceae | Xanthobacteraceae |
| Carnobacteriaceae | Carnobacteriaceae | Hyphomonadaceae |
| Barnesiellaceae | Barnesiellaceae | Isosphaeraceae |
| Rikenellaceae | Rikenellaceae | Aerococcaceae |
| RB40 | RB40 | Burkholderiaceae |
| Alicyclobacillaceae | Alicyclobacillaceae | Haliangiaceae |
| Sporichthyaceae | Sporichthyaceae | Barnesiellaceae |
| Christensenellaceae | Christensenellaceae | Phormidiaceae |
| Legionellaceae | Legionellaceae | Trueperaceae |
| Mogibacteriaceae | Mogibacteriaceae | Rs-045 |
| Peptococcaceae | Peptococcaceae | Mogibacteriaceae |
| Xanthobacteraceae | Xanthobacteraceae | Intrasporangiaceae |
| Cellulomonadaceae | Cellulomonadaceae | Dermacoccaceae |
| Exiguobacteraceae | Exiguobacteraceae | Streptomycetaceae |
| Haliangiaceae | Haliangiaceae | Williamsiaceae |
| Erythrobacteraceae | Erythrobacteraceae | AKIW874 |
| Aurantimonadaceae | Aurantimonadaceae | Dermatophilaceae |
| Pseudonocardiaceae | Pseudonocardiaceae | Carnobacteriaceae |
| Leuconostocaceae | Leuconostocaceae | Nostocaceae |
| Methylophilaceae | Methylophilaceae | Legionellaceae |
| Synergistaceae | Synergistaceae | Bacteriovoracaceae |
| F16 | F16 | Exiguobacteraceae |
| Nocardiaceae | Nocardiaceae | Cellulomonadaceae |
|  |  | Dietziaceae |
|  |  | Synergistaceae |
|  |  | Listeriaceae |
|  |  | Rhodobiaceae |
|  |  | F16 |
|  |  | HTCC2188 |
|  |  | Frankiaceae |
|  |  | Peptococcaceae |
|  |  | Geodermatophilaceae |
|  |  | Microthrixaceae |
|  |  | Methylophilaceae |
|  |  | Alteromonadaceae |
|  |  | Erythrobacteraceae |
|  |  | Aurantimonadaceae |
|  |  | Sporichthyaceae |
|  |  | Leuconostocaceae |
|  |  | Nocardiaceae |
|  |  | Planococcaceae |
